# Inferring room-level use of domestic space heating from room temperature and humidity measurements using a deep, dilated convolutional network

**DOI:** 10.1016/j.mex.2021.101367

**Published:** 2021-04-27

**Authors:** Niklas Berliner, Martin Pullinger, Nigel Goddard

**Affiliations:** The University of Edinburgh, School of Informatics, Informatics Forum, 10 Crichton Street, Edinburgh, EH8 9AB, United Kingdom

**Keywords:** Residential heating behaviour, Inference, Machine learning, Ambient temperature and humidity

## Abstract

Time series data about when heating is on and off in homes can be useful for research on building energy use and occupant behaviours, particularly data at room level and at a granularity of minutes. Direct methods which measure the temperature of radiators and other heaters can be effective at producing such data, but are expensive. Indirect methods, which infer heating on- and off-times from ambient room temperature data, can be cheaper but produce more error-prone data. Existing indirect methods have however utilised relatively simple prediction algorithms based on changes in ambient temperature between closely adjacent time points. In the method presented here we have implemented several refinements to this approach:

• An Artificial Neural Network algorithm is applied to the prediction task: a deep, dilated convolutional network.
• A wider range of input features is utilised to base predictions upon: ambient room temperature and humidity, and external temperature and humidity.
• Predictions for each time point are based on data from a wider, 600-minute, time window.
• We evaluate model performance on a dataset with 10 min granularity and achieve mean precision and recall during the heating season of >=0.74 for individual time points, and >=0.82 for full heating events, outperforming comparator methods.

## Graphical abstract

**Figure:**
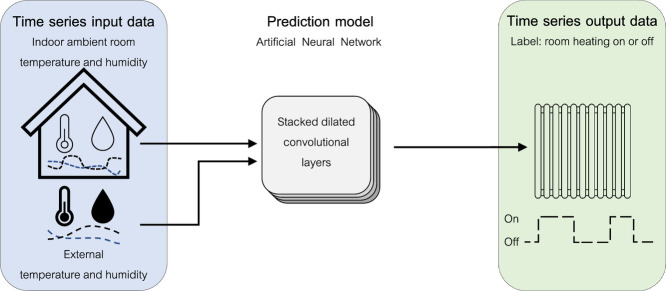

**Table:** Specifications Table

Subject Area	Energy
More specific subject area	Domestic heating use
Method name	Inference of domestic heating use using an artificial neural network
Name and reference of original method	Huebner, G. M., et al. “The Reality of English Living Rooms – A Comparison of Internal Temperatures against Common Model Assumptions”. In: Energy and Buildings 66 (2013), pp. 688–696 [1]
Resource availability	Method is implemented in Python using standard data science libraries.

*Method details

## Overview

This paper presents a new Machine Learning methodology for inferring domestic radiator use. The method outputs times series data indicating whether a room's space heating is on or off at each time point. Inputs comprise sensor data on the room's ambient temperature and humidity, and secondary data on external temperature and humidity. This paper describes the methodology, a deep, dilated convolutional network, and presents an evaluation of its performance in inferring when room radiators are on or off, using a recently published household energy use dataset. The method is adapted for input and output data at a 10 min granularity, although could in principle be adapted to function with data of different granularity.

Room-level time series data on when space heating is used in homes has a range of potential end uses, such as for assessing how occupant heating behaviours contribute to building energy use, and how this varies by time of day, day of week, or season. In one study that compared five approaches to generating data on heating on-times (for a home's central heating as a whole, rather than for individual rooms) [2], direct sensor measurement of radiator temperatures was most effective; however the authors note that such data are harder and more costly to obtain than indirect approaches. Indirect approaches using ambient room temperatures (3 methods) and gas meter data (1 method) to infer heating on-times were less reliable, particularly in warmer months, but use more readily available data. It should be noted that the prediction models using room temperatures were relatively simple, based on a change in temperature between time points (with 45 min input data). In one method this was between adjacent time points, whilst in two others this was based on cumulative change over several time points to account for short-term state changes controlled by thermostatic controls.

The methodology presented in this paper builds on a method described and evaluated in [1], which is also evaluated in [2], that uses room temperature to identify on-times for the entire central heating. In their work, the authors determine the state of the heating system by investigating the relative temperature change between multiple time points. If a continuous increase (or decrease) in temperature for 7 time points was observed which surpassed the threshold value, it was assumed that the heating system changed to *on* (or *off*, respectively).

We extend beyond that work in the following ways: we focus on room-level rather than home-level granularity; we apply more advanced ML methods rather than rules-based methods; we utilise a wider range of input features to base predictions upon.

The method described here is a deep, dilated convolutional network, a form of artificial neural network (ANN). ANNs have been applied in various applications relating to energy use in buildings [3]. ANNs were successfully used to predict indoor temperature [4,5] and applied to determine optimal heating start times in buildings [6]. However, to the best of our knowledge, no previous work has used ANNs to predict space heating use in rooms based on indoor temperature and humidity data.

## Method

In this section we describe our deep, dilated convolutional network method for inferring room-level domestic heating use, using room and external temperature and humidity time series data as inputs, and outputting predictions of whether the heating is on or off at each time point. Deep convolutional networks, originally developed for visual imagery, have proven to be a powerful tool for time series data analysis [7]. Instead of the two spatial dimensions characterising images, the methods are adapted for the one time dimension of such time series data. Dilated convolution is a technique to increase the receptive field of convolutional neural networks without loss of resolution or coverage [8]. Stacking dilated convolutional layers produces exponentially increasing receptive field sizes while remaining computationally efficient, which makes the approach a good candidate architecture for models aiming to combine long- and short-range effects. The long and variable lag, between radiator heating and room temperature and humidity responding to the radiator being on, suggested that such an architecture could perform well for the current prediction task.

### Demonstration dataset

In this paper, the authors demonstrate the methodology and evaluate its performance using data from the IDEAL Household Energy Dataset [9]. The dataset includes room temperature and humidity data collected using wall-mounted digital sensors from 255 homes in and around Edinburgh, UK, and external temperature and humidity data from local weather stations, provided by a secondary weather data service. 39 of these homes, which are the ones used for this demonstration, also had radiator temperature sensors installed, fitted to the radiator inflow and outflow pipes of radiators in each room. Such probes might underestimate the true pipe temperature depending on the quality of their attachment, but can nevertheless be taken as a measure of the surface temperature of the radiator body: the measured temperature will start to rise once hot water is pumped into the radiator, and will slowly return to room temperature once the supply of hot water stops. The rate of temperature decrease might vary between radiators, possibly introducing some variations in the expected lag between the stop of hot water flow into the radiator and our assumed switch from radiator *on* to *off*.

The 39 homes have the full range of data used in the evaluation presented in this paper for durations of between 23 and 175 days, with a mean of 80 days, over a 23 month period ending in June 2018.

All room sensors reported readings at 12 s intervals. For this study, sensor data were downsampled to a 10 min granularity by taking the mean of the reported values. If no value was reported during the 10 min period we set the value to NaN, i.e. missing. Weather data were reported at 15 min intervals. For this study, we upsampled these to the same 10 min granularity by taking the nearest timestamped datapoint, meaning the value was either from the same timepoint or was from five minutes before or after. In all cases, missing data were then filled if they were within 3 readings forwards or backwards of a non-missing data point. Imputed values were computed by linear interpolation between the readings immediately before and after a gap. Thus mid-sequence gaps of up to six time points (60 min) were completely filled, while larger gaps had three imputed values at each end (30 min at each end) while retaining missing data elsewhere. (Note that missing values at the very beginning or end of a data series were imputed by copying the first (or last) non-missing reading backwards (or forwards) up to a maximum of three time points).

The indoor and outdoor temperature and humidity data were then combined, resulting in one time series with four features per room.

### Ground truth labels

Our model is designed to solve the classification problem of predicting if space heating was *on* or *off* at each time period, and in this evaluation was trained specifically for radiators being on or off. Ground truth labels for the radiator being on or off were generated based on the radiator pipe probe temperatures. Following our reference method [1], we defined a radiator to be *on* if its temperature was above room temperature. In our study, we took this to be by 5 °C or more. Specifically, if input and output pipe temperatures were available, we used the average between both as a proxy for the radiator temperature. If the output pipe temperature was missing, we used the input temperature alone, and vice-versa. If no input temperature was available for a time point, the temperature was recorded as missing (with gaps interpolated where possible as described above).

This approach captures periods when the radiator is substantially hotter than the ambient room temperature, and as such is radiating heat into the room, actively warming it. This differs to some extent from when the central heating boiler that heats the radiator is on. Firstly, when the central heating is ‘on’, it nevertheless may cycle through short periods when hot water is not being supplied, whenever the water returning to the boiler is above a certain temperature or a central thermostat detects a setpoint ambient room temperature has been reached. Secondly, when the central heating is turned ‘off’ again (e.g. manually or by a timer), the radiators remain hot for a period as the hot water within them cools down, radiating the remaining heat into the room.

### Network architecture

The method described here used a rolling window approach with a step size of 1 to split the time series for each room into smaller segments. Segments with missing data were discarded from further analysis. The length of the segments was set to 60, corresponding to a window width of 600 min. A sequence-to-sequence approach was used, with the output width set to the same value as the input. We used a sigmoid activation function as the last layer of our model, which outputs values between zero and one which can be treated as “probablities” of the radiator being on. Using a step size of 1 results in overlapping predictions, which were combined by taking the average of all predicted probabilities. The resulting probabilities were binarised using a threshold of 0.4, i.e. every time point with a computed probability of 0.4 or less was classified as having the radiator turned off; while every time point with a probability above 0.4 was classified as having the radiator turned on.

We selected the threshold of 0.4 as this was the value that resulted in approximately equal *per-bin* precision and recall on the full validation set. A higher threshold would lead to higher precision at the expense of diminished recall, and vice versa. In real-world use cases, the threshold could therefore be adjusted depending on the relative importance of achieving fewer false positives versus fewer false negatives.

Batch normalisation [10] was applied after each convolution and dense layer respectively and before each activation function. For the activation function, the Parametric Rectified Linear Unit (PReLU), a generalisation of the Rectified Linear Unit (ReLu) was chosen [11]. The full network architecture is described in Table 1.

**Table 1:** Layers used in the ANN model. The dilation rate is increased subsequently to increase the depth of field. Each layer except the output layer is followed by a batch normalisation layer and a Parametric Rectified Linear Unit (PreLU) as activation function. The final layer uses the sigmoid activation function to predict a probability of the radiator being on at each time step.

	Layer Type	Size	Kernel Size	Padding	Dilation Rate
Input Layer	Conv2D	16	3 × 4	same	1 × 1
	Conv2D	8	3 × 4	same	1 × 1
	Conv2D	4	3 × 4	same	2 × 1
	Conv2D	4	3 × 4	same	3 × 1
	Conv2D	4	3 × 4	same	4 × 1
	Conv2D	4	3 × 4	same	1 × 1
	Conv2D	4	3 × 4	same	1 × 1
	Conv2D	2	1 × 4	valid	1 × 1
	Dense	512	–	–	–
	Dense	512	–	–	–
Output Layer	Dense	60	–	–	–

### Model training and validation

The network was implemented using the Keras framework [12] and trained using the Adam optimiser [13] with binary cross-entropy loss. The model was trained for 100 epochs, and the initial learning rate of 1e-06 was reduced by a factor of 0.8 if no improvement in validation loss was observed for 3 epochs, to a minimum of 1e-07.

To obtain a robust estimate of the model performance, we split the training data on homes and performed 10-fold cross validation. Each cross validation split was used to predict its respective validation data and these predictions were then combined to obtain predictions for the entire dataset. By splitting the data based on homes instead of splitting rooms directly, each model is validated on homes which it did not see during training. This should give a better estimate of the generalisability of the network. We used random search to split the homes into 10 groups, minimising the maximum difference in the amount of training data between groups.

### Weight initialisation

The importance of choosing an appropriate weight initialisation technique for training neural networks is widely recognised [14]. Yu and Koltun [8] noticed that their network of dilated convolutions failed to improve model accuracy with standard weight initialisation. Instead, they utilised a form of identity initialisation in which each layer passes the input directly to the next. Batch Normalisation, a common technique to improve training of neural networks, is known to reduce dependence on weight initialisation and improve generalisation [10,15]. Based on our testing, the proposed identity-like initialisation did not improve training over standard He initialisation [11]. This could be due to the Batch Normalisation layers in our architecture, which were not used in [8]. As consequence, we used He weight initialisation in all layers.

## Evaluation of model performance

This section provides an evaluation of the performance of our model using data from the IDEAL Home Energy Dataset, which was described earlier.

### Approach to model evaluation

We evaluated the model performance using precision and recall, standard score functions for such classifiers. Precision provides an indication of the level of false positives, being the proportion of cases that the model predicts to be positive that were truly positive, given by Eq. (1). Here, a score of 1 indicates no false positives; a score of 0 indicates only false positives. Recall provides an indication of the level of false negatives, being the proportion of positive cases that the model classified correctly, given by Eq. (2). Here, a score of 1 indicates no false negatives; a score of 0 indicates only false negatives. In the equations, TP = True Positives, FP = False Positives, and FN = False Negatives.

(1) $$Precision:\frac{\text{TP}}{\text{TP}\;+\;\text{FP}}$$

(2) $$Recall:\frac{\text{TP}}{\text{TP}\;+\;\text{FN}}$$

**Fig. 1:**
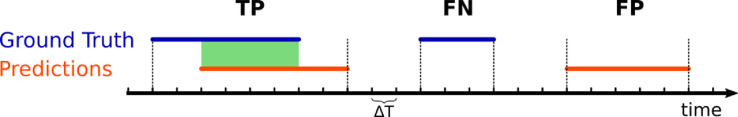
Classification of predictions on a *per-event* basis. Each model works on evenly spaced time steps, resulting in one prediction per time step ∆*T*. Next to the classification performance per ∆*T*, we evaluate the performance per heating event. In this context, a True Positive (TP) event is understood as any event with overlap between Ground Truth and Predictions. True heating events which are missed by the model are identified as False Negatives (FN) and predictions without Ground Truth are denoted as False Positives (FP) respectively.

Precision and recall were computed for two use-cases. First, they were computed on a *per-bin* basis, i.e. a classification task where each bin (each 10-minute time step) was treated independently of each other. Second, they were computed on a *per-event* basis. Since we are predicting “radiator on” events, it is useful to understand how well the model recovers these heating *events*. Here *events* are understood as “the radiator was on” irrespective of the duration. This metric includes the assumption that the time steps are not fully independent of each other. Instead, a radiator will be *on* or *off* for substantially longer than one time step. The definition of True Positives, False Negatives, and False Positives understood *per-event* is illustrated in Fig. 1.

### Baseline models

To compare the performance of the ANN model, two baseline models are used. The first model, *baseline sampling*, first computes a probability of the radiator being *on* per month. This probability is estimated by computing the number of bins the radiator was on divided by the total number of observed bins for each month. Predictions are then made by sampling from a binomial distribution. The second model, *baseline regression*, uses the same four input features as the ANN model, ambient room temperature and humidity and external temperature and humidity, to predict the probability of the radiator being *on* per bin using logistic regression. As opposed to the ANN model, the logistic regression was not trained on an input window of multiple time steps, but instead used each time step as individual input/output pairs. We used the logistic regression implementation of *scikit learn* [16] with default initialisation parameters.

### Model performance

Our model reached an overall *per-bin* precision and recall of 0.73 and 0.75 respectively. The *per-event* performance was 0.80 and 0.75 for precision and recall respectively. Our model showed performance variation across the year, performing worse during summer months. During summer, very few heating events were observed, likely rendering it difficult for the model to distinguish between *on* and *off* events. Table 2 summarises the achieved precision and recall for the baseline models and our ANN model respectively. While the sampling-based baseline performs poorly on a *per-bin* basis, it reaches a *per-event* precision and recall of 0.08 and 0.82 respectively. Since this model draws its predictions per time step from a binomial distribution, it is to be expected that no temporal information is available, resulting in predictions of many scattered bins being on. The low precision *per-event* is a result of these many “scattered” predictions, many of which are counted as False Positives. The same “scattered” predictions result in a very high chance of correctly predicting at least one time step per true heating event (cf. Fig. 1 for an explanation on how the events are counted). Whilst the regression-based model also does not include temporal information, it is nevertheless able to avoid “scattered” predictions.

**Table 2:** Precision and recall on *per-bin* and *per-event* basis. The heating period is the months November, December, January, February and March; the no-heating period comprises May, June, July, August and September; April and October form the transition periods.

	*per-bin*		*per-event*	
	Precision	Recall	Precision	Recall
Baseline sampling	0.25	0.26	0.08	**0.82**
Baseline regression	0.55	0.17	0.74	0.12
ANN Overall	0.73	0.75	0.80	0.75
ANN Heating Period	**0.74**	**0.81**	**0.83**	**0.82**
ANN Transition Periods	0.72	0.67	0.79	0.70
ANN No-Heating Period	0.64	0.47	0.65	0.49

**Fig. 2:**
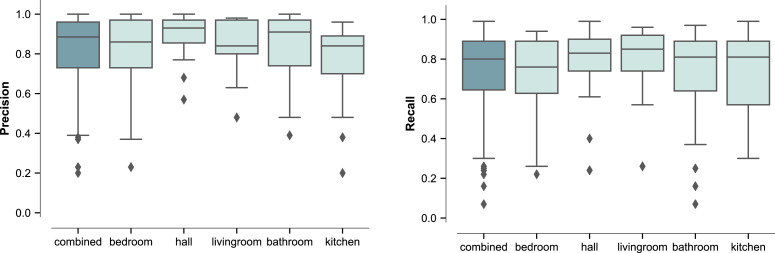
Boxplots of precision and recall of the ANN model for prediction of radiator use *per-bin*, for rooms with 50 or more true heating events, across all time periods. The boxplots on the left of each chart show the combined distribution across all room types.

It is of value for understanding the method's performance to describe the *per-bin* distribution of precision and recall across rooms. While the performance is consistent for the majority of rooms, the results for some of the rooms deviate strongly. Fig. 2 summarises the respective distributions for rooms with at least 50 or more observed true radiator-on *events*. While precision and recall for the majority of rooms lie above 0.8, there are exceptions observed. Our model performs generally worse for kitchens. Our ground truth is a measure of radiator use, whilst in kitchens, non-radiator-related events like cooking would also be expected to make the prediction input features, humidity and temperature, fluctuate strongly. The same holds true for bathrooms, although we find that the model tended to achieve higher precision and, to a lesser extent, recall, for bathrooms than for kitchens. This suggests that our model may be better able to distinguish between non-radiator-related events in bathrooms than in kitchens.

**Fig. 3:**
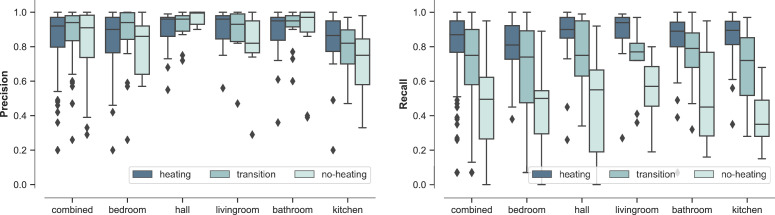
Boxplots of precision and recall of the ANN model for prediction of radiator use *per-bin*, for rooms with 50 or more true heating events, showing performance for the heating period (November-March), transition periods (April and October) and non-heating period (May-September). The boxplots on the left of each chart show the combined distribution across all room types.

We furthermore investigated the distributions of precision and recall for each room type broken down by the same periods of the year used in Table 2, i.e. for the heating period (November-March), transition periods (April and October) and no-heating period (May-September). Fig. 3 summarises the distributions for rooms with at least 50 or more observed true radiator-on *events*. These provide further detail behind the overall results from Table 2 and Fig. 2. For both precision and recall, they indicate that the strong model performance in the heating season is largely consistent between room types. For precision, the figure shows that behind the fairly small overall decline in model performance presented in Table 2 moving from the heating period, through the transition period and into the non-heating period, there is some variation between room types in the level of decline; however, in none is it very large. For recall meanwhile, the more substantial decline in performance between those periods is relatively consistent across room types. The figure also highlights that the poorer performance observed for kitchens compared to other room types is apparent across the year, and is not just focused on one part of the year.

By investigating the distribution of heating event durations for True Positive and False Negative predictions respectively, we found that heating events of shorter than approximately 60 min are very likely to be missed by our model (data not shown). We suspect that the ambient conditions of a room typically need around one hour to respond sufficiently to a radiator being turned on for it to be reliably detected. We further speculate that the low recall rate observed for some bedrooms might be due to short “bursts” of heating, which we found various examples of in the demonstration dataset when manually inspecting the results. In particular, the bedroom with a recall near zero had many short bursts of radiator use, in which the radiator was not even recorded as reaching close to full temperature.

One source of error leading to a large number of False Positives (i.e. low precision) could be additional heat sources, such as electric radiators, open fires, heat transfer from other rooms through open doors and heating from direct sunlight entering the room. Manually inspecting the results, we think that for at least one room, actual heating in an adjacent room was picked up by the model. These forms of “miscellaneous” heating sources might partly contribute to low precision in some rooms, and highlight that our model detects heating of any kind, whereas the labels used with the demonstration dataset are exclusively for radiator use.

As the purpose of the model is to predict heating patterns, it is further important to understand how well it recovers the duration of individual heating events. We find that in 78% of the cases a prediction is made, the predicted duration is within one hour of the true heating duration (data not shown). This indicates the performance in predicting heating event durations in absolute terms. In relative terms, we find that our model achieves a relative difference between predicted durations and true heating durations of within ±50% in 81% of the cases (data not shown). Here, a value of −50% relative difference indicates that the predicted duration is only half of the true duration, while a value of +50% indicates that the predicted duration is one and a half times the true duration.

**Fig. 4:**
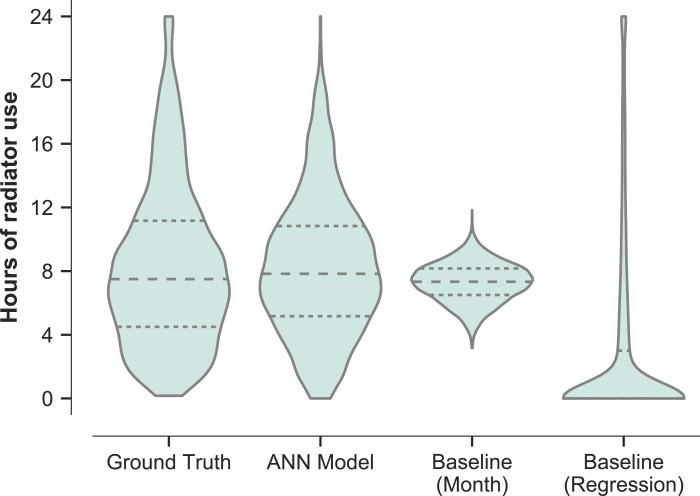
Distribution of heating durations (hours of radiator use) per day. The observed and predicted durations during the heating season (beginning of November to end of March) is computed based on the 10 min window data. Only data from days with true heating events are included. The dashed lines show the quartiles of the distributions.

Another value of applied interest is the per-day duration of radiator on-time per room, particularly during the heating season. This might be easier to predict, as only information about the daily usage is required and no details about when the heating is turned on is needed. We computed the duration for which radiators were on per day based on the 10 min ground truth from the demonstration dataset and compared it to the durations predicted by the ANN model and the baseline models, focusing on the heating season. In Fig. 4, the distribution of the number of hours that radiators were on per day is shown, summarised across all rooms and homes. It can be seen that the baseline sampling method is able to predict the average duration, but fails to predict both the large proportion of days with short heating durations and the long tail of days with longer durations. The regression based model meanwhile accounts for some of the long tail while severely underestimating the average heating duration. The ANN model greatly outperforms both of the baseline models, giving a good prediction of the average value, the quartiles and the overall distribution, with some underestimation of the proportion of days with short and long heating durations.

## Conclusion

This paper describes a new Machine Learning methodology for producing estimates of on and off times for room space heating using room and outdoor temperature and humidity readings. The evaluation, based on a sample of 39 homes from the Edinburgh region of the UK from the IDEAL Household Energy Dataset [9], indicates that it achieves a good level of precision and recall in a residential setting, particularly during the heating season and especially for full heating events, and that during the heating season it provides a good estimate of the distribution of daily heating durations across rooms and days. Performance is reduced in kitchens and bathrooms, likely due to non-radiator-based factors such as cooking and washing activities that affect ambient temperature and humidity.

The model in this case was trained and evaluated on a dataset that contained labelled data only for radiator on and off times. However, it is likely that it would work similarly well for many other forms of on/off space heating technology, such as individual gas fires or electric heaters, perhaps with the exception of technologies such as underfloor heating that tend to be on at a lower temperature for longer periods. Adaptation for use in non-residential settings might also be successful, but is likely to depend on the setting, e.g. room characteristics such as floor area and ceiling height.

The method should also be adaptable to data with sampling frequencies other than the 10 min frequency it is currently tailored for. It would require adaptations to aspects of the model design, notably the input window size. Experimentation with parameters and training and validation on the new dataset would be required.

Our approach expands the opportunities to produce data on space heating patterns, which could be of value for future research or other purposes. Work using such a method would need to be mindful of the increased level of ‘noise’ (false negatives and false positives) in the resultant dataset on radiator on-times compared to direct radiator measurements, and balance this against the likely benefits in terms of being able to use more readily and/or cost-effectively obtainable input data.

## Declaration of Competing Interest

The authors declare that they have no known competing financial interests or personal relationships that could have appeared to influence the work reported in this paper.
